# Peripheral and central macrophages in obesity

**DOI:** 10.3389/fendo.2023.1232171

**Published:** 2023-08-31

**Authors:** Sayani Mukherjee, Silje Skrede, Martha Haugstøyl, Miguel López, Johan Fernø

**Affiliations:** ^1^ Hormone Laboratory, Department of Medical Biochemistry and Pharmacology, Haukeland University Hospital, Bergen, Norway; ^2^ Mohn Center for Diabetes Precision Medicine, Department of Clinical Science, University of Bergen, Bergen, Norway; ^3^ Department of Physiology, CIMUS, University of Santiago de Compostela, Santiago de Compostela, Spain; ^4^ CIBER Fisiopatología de la Obesidad y Nutrición (CIBERobn), Santiago de Compostela, Spain; ^5^ Department of Clinical Science, Faculty of Medicine, University of Bergen, Bergen, Norway; ^6^ Department of Medical Biochemistry and Pharmacology, Haukeland University Hospital, Bergen, Norway

**Keywords:** obesity; inflammation, macrophages, adipose tissue, microglia, hypothalamus, small extracellular vesicles

## Abstract

Obesity is associated with chronic, low-grade inflammation. Excessive nutrient intake causes adipose tissue expansion, which may in turn cause cellular stress that triggers infiltration of pro-inflammatory immune cells from the circulation as well as activation of cells that are residing in the adipose tissue. In particular, the adipose tissue macrophages (ATMs) are important in the pathogenesis of obesity. A pro-inflammatory activation is also found in other organs which are important for energy metabolism, such as the liver, muscle and the pancreas, which may stimulate the development of obesity-related co-morbidities, including insulin resistance, type 2 diabetes (T2D), cardiovascular disease (CVD) and non-alcoholic fatty liver disease (NAFLD). Interestingly, it is now clear that obesity-induced pro-inflammatory signaling also occurs in the central nervous system (CNS), and that pro-inflammatory activation of immune cells in the brain may be involved in appetite dysregulation and metabolic disturbances in obesity. More recently, it has become evident that microglia, the resident macrophages of the CNS that drive neuroinflammation, may also be activated in obesity and can be relevant for regulation of hypothalamic feeding circuits. In this review, we focus on the action of peripheral and central macrophages and their potential roles in metabolic disease, and how macrophages interact with other immune cells to promote inflammation during obesity.

## Introduction

The increasing prevalence of obesity represents a serious health concern in both developed and developing countries. Obesity is associated with several co-morbidities, such as type 2 diabetes (T2D), atherosclerosis, non-alcoholic fatty liver disease (NAFLD) and cancer, and is estimated to be responsible for the deaths of more than 4 million people each year (1). Inflammation is believed to be important in the pathogenesis of obesity and its co-morbidities. Indeed, dysregulation of immune cells located in metabolically active tissues, in particular in the adipose tissue (AT), has been shown to contribute to disease progression. A plethora of immune cells may contribute to a pro-inflammatory state in obesity, but macrophages seem to be of special importance, representing up to 50% of the immune cells in the obese AT (2). In recent years it has become clear that obesity-induced inflammation also occurs in the central nervous system (CNS) (3), which may contribute to the metabolic phenotype as well as to CNS-specific pathologies associated with obesity, including neurodegenerative diseases like Alzheimer’s disease and Parkinson’s disease (4, 5). However, how peripheral and central inflammation is connected in obesity remains unclear. In this review we focus on macrophages in metabolically active tissues and their role in metabolic regulation, and we examine how central and peripheral immune cells interact during obesity.

Macrophages are part of the innate immune system, and together with other innate immune cells, such as neutrophils, eosinophils, and natural killer (NK) cells, they form the first line of immune defense that respond quickly to foreign invaders. The adaptive immune system, on the other hand, elicits a slower response, but has the ability to generate an immunologic memory towards specific pathogens and to trigger effective immune responses when exposed to the same pathogens in the future (6). Accumulating evidence indicates that the innate immune system may also form such a long-term memory after encountering a stimulus, a concept termed “trained immunity” that involves sustained epigenetic reprogramming that potentiates inflammatory responses to future challenges (7–9). Obesity has been shown to promote such trained immunity in innate immune cells, where for example obesity-induced changes in AT macrophages (ATMs) were maintained after weight loss in mice (10), and elevated free fatty acids induced a sustained inflammatory phenotype in myeloid cells *in vitro* (11).

Innate and adaptive immune cells can be categorized as either circulating or tissue-resident cells. Circulating immune cells are those who are transported in the blood stream, recognizing and killing infected/abnormal cells. Examples of circulating innate immune cells are monocytes and macrophages, whereas T cells and B cells represent circulating adaptive immune cells (12, 13). Tissue-resident immune cells play integral roles at all stages of the immune response. It should be noted that in addition to responding to infectious challenges and mediating the resolution of inflammation, these cells also have important roles in maintaining tissue homeostasis and repair (10, 14). Tissue-resident immune cells are found in AT, liver, pancreas, brain and intestine, where they express specific surface markers depending on the tissue that they reside in (15–17) and the functions they exert. Examples of major tissue-resident immune cells are ATMs, hepatic Kupffer cells (KC) and CNS microglia.

In recent years, there has been increased awareness of the importance of ATMs in obesity. Adiposity has been shown to affect their abundance, subset ratios, and phenotype diversity, which can contribute to alterations in whole body metabolism. As a response to specific microenvironmental stimuli, including metabolic stress signals such as fatty acids and glucose, levels of proinflammatory M1-like ATM subtypes are elevated, whereas alternatively activated M2-like macrophages, with anti-inflammatory properties, are reduced (18). The M1-like ATMs have been shown to impair insulin signaling in both mice and humans (19). In contrast, the M2-like ATMs are believed to mediate protection from these metabolic disturbances and to play a role in AT homeostatic functions (20, 21). Various subcategories of M2 macrophages are found, including M2a, M2b, M2c, and M2d (22). Furthermore, it is now well established that there exists a complex range of ATM phenotypes that display characteristics differing from the classical M1- and M2-like ATMs (21, 23, 24). These cells differ in their expression of cell surface markers, cytokine secretion profile, and biological characteristics, which is described in more detail below.

Although ATMs are considered the most important immune cells that mediate metabolic (dys)regulation during obesity, other cells such as T cells and NK cells are also of importance. This is partly due to their role in modulating ATM polarization, emphasizing that the interaction between immune cells plays a significant role in inflammation in obese mice and in people with obesity (25–29). In this review we aim to describe how immune cells, especially macrophages, are involved in obesity-related metabolic inflammation, and to illustrate how the balance between pro- and anti-inflammatory immune cells is important to maintain a healthy tissue homeostasis during changes in nutritional status.

## Macrophage polarization and inflammation in the adipose tissue during obesity

Obesity is a consequence of excess nutrient intake and a positive energy balance that increases both the size of mature adipocytes (hypertrophy) and the number of adipocytes by *de novo* formation from preadipocytes (hyperplasia) (30). Fatty acid-binding protein (FABP4/aP2) has been shown to be important in controlling adipocyte size, differentiation and the recruitment of new adipocytes (31). Further, it regulates mitochondrial redox signaling through uncoupling protein 2 (in contrast to UCP1, which controls energy expenditure, UCP2 mainly controls ADP/ATP ratio) (32) in a process commonly termed “adipose tissue remodeling”, which is important to adapt to the increased energy load. However, during obesity, hypertrophy may not be compensated by sufficient blood vessel vascularization, which may lead to hypoxia, mitochondrial dysfunction and, subsequently, AT stress. In turn, this can lead to an increase in pro-inflammatory responses. Normally, the AT immune cells play vital roles in maintaining AT homeostasis during tissue remodeling, but pro-inflammatory activation over time may lead to chronic inflammation and the activation of reactive super oxygen species (ROS), impairing AT hormonal signaling and function (33).

In hypoxic AT, anaerobic glucose breakdown may fuel pro-inflammatory processes via activation of hypoxia-inducible factor 1-alpha (HIF-1a) (34). Moreover, the hypoxic signal has been shown to inhibit the mitochondrial electron transport chain and decrease oxidative phosphorylation (OXPHOS), leading to increased ROS production and a shift towards glycolytic processes that may further stimulate the inflammatory phenotype (35). The activation of HIF-1a also contributes to direct regulation of innate and adaptive immune cells in the AT, including epithelial cells, neutrophils, macrophages, dendritic cells (DC), T cells, B cells, NK cells and innate lymphoid cells (36). For macrophages, the elevated glycolysis leads to a shift towards M1-like ATM, as well as increased expression of genes involved in macrophage adhesion and inhibition of macrophage migration from the hypoxic AT (37, 38). It has also been reported that obesity activates the FABP4/UCP2 axis in macrophages, resulting in the activation of inflammasomes (32). Further, the activation of toll-like receptors (TLRs) by elevated fatty acids during obesity is essential for the capacity of ATMs to generate pro-inflammatory cytokines, such as tumor necrosis factor alpha (TNFa) and interleukin (IL)-6 (39). This is mediated probably through the activation of pathways regulated by the nuclear factor kappa B/c-Jun N-terminal kinases (NFκB/JNK) and Inhibitory kappa B kinase (IκB) inhibitor (40, 41). The M2-like ATMs rely on mitochondrial OXPHOS and are thus reduced under these conditions, rendering the hypoxic environment in the expanding AT supportive of an elevated M1/M2 ratio (18, 42).

All these studies suggest that expansion of AT during obesity can trigger adipocyte hypertrophy and hypoxia, which finally may lead to chronic, low-grade inflammation where ATM activity and polarization play an important role.

## Adipose tissue macrophage heterogeneity

In AT of both animals and people with obesity, pro-inflammatory macrophages form so-called crown-like structures (CLS), surrounding dead or dying adipocytes, in particular evident in the visceral AT (VAT) (43, 44). In recent years it has become clear that there are numerous types of ATMs that can be characterized based on the combination of surface proteins they express. Also, recent advances in single-cell sequencing have demonstrated that a variety of macrophage subsets exist that differ from the conventional M1-/M2-like macrophages, some of them exhibiting proinflammatory characteristics, while others are more metabolically active (23, 24, 45). In humans, CLS macrophages have been shown to express the surface proteins CD206 and CD11c (46), and are associated with systemic markers of metabolic dysfunction, supporting the idea that pro-inflammatory ATMs may have unfavorable effects on metabolic health (45–48). However, more recent literature has claimed that CLS are indispensable in controlling metabolic homeostasis, and that their accumulation around dying adipocytes is a mechanism to get rid of excessive adipocyte lipids in a non-harmful way (49–51). Nevertheless, the excessive inflammation associated with CLS can result in “collateral damage”, where the effect on metabolism depends on the type of pro-inflammatory and/or metabolically active ATMs that predominate in the AT. Interestingly, pro-inflammatory ATMs does not seem to express the classical surface markers of activated M1 macrophages, CD38, CD319, and CD274 (18). Instead, in addition to the combination of CD206 and CD11c as described above, ATMs have been shown to express ABCA1, CD36, and PLIN2, defining so-called metabolically activated macrophages (MMe) (18). These cells promote the clearance of dead adipocytes via lysosomal exocytosis (51). NADPH oxidase 2 (NOX2) was shown to be a major mediator of the inflammatory and adipocyte-clearing properties of MMe macrophages, and *Nox2* ^−/−^ mice exhibited insulin resistance, liver inflammation, and visceral lipoatrophy, characterized by deposition of dead adipocytes and dysfunctional ATM lysosomal exocytosis (51). In another study, mouse Ly6c-expressing ATMs have been shown to be predominant outside of CLS and to display adipogenic properties, while CD9-lipid associated ATMs (LAMs) were found to be present within CLS of HFD-fed mice and humans with obesity (50). The LAMs expressed “triggering receptor expressed on myeloid cell 2” (TREM2) lysosomal acid lipase, and controlled lipid metabolism and phagocytosis in mouse AT (50). Interestingly, TREM2 deletion has been shown to cause weight gain, hypercholesterolemia, and glucose intolerance in mice due to the loss of LAM function, lipid uptake, and storage (14), indicating that Trem2+ LAM cells may be key in the mitigation of metabolic disruption in the AT. Other ATMs that have been described are iron rich (MFe) macrophages, shown to have an inflammatory phenotype in obese mice (52), and antioxidant macrophages (Mox) ATMs, essential to iron and oxidative stress handling (53). In agreement with findings described above (46), a recent study from our laboratory found a positive correlation between the level of insulin resistance in 80 patients with obesity and the pro-inflammatory ATM ratio between M1-like (CD206/CD11c expressing cells) and M2-like (CD206 expressing cells) in both VAT and subcutaneous AT (SAT) (29). However, using a surface proteomics approach we also identified several new surface markers on these immune cells, indicating that a plethora of M1-like and M2-like ATM subtypes exist in the human AT (54). According to these findings, it is obvious that ATMs exist in pro- and anti-inflammatory forms, but in the light of recent findings the M1/M2 dichotomy is gradually replaced by a more differentiated view on ATM characteristics. ATMs act in response to obesity in a temporal and site-specific manner that may be both beneficial and harmful.

## Other immune cells in the adipose tissue that interact with adipose tissue macrophages

The details of how the different ATM subtype’s function and act in response to metabolic stimuli is further complicated by their interaction with other adipose-resident immune cells, such as T cells, invariant natural killer T (iNKT) cells, gamma delta (γδ) T cells, NK cells, DCs, eosinophils and their diverse phenotypes that may manifest in the obese AT. T cells are the second most prevalent immune cells in AT. It has been reported that in obesity there is an increased frequency of pro-inflammatory CD4+ T cells (e.g., Th1 and Th17 cells), and cytotoxic CD8+ T cells, and reduction in anti-inflammatory CD4+ T cells (e.g., Th2 cells) and FOXp3 regulatory T cells (Tregs) (55). The Th1 cells and CD8+ T cells have been shown to stimulate M1 ATM polarization in obese mice AT through IFNg production. There may, however, be several subgroups of AT-T cells with potentially different metabolic effects. An in-depth analysis of T cells in human AT depots has been conducted by our laboratory. Here, distinct subsets of T cells were shown to express markers such as CD26 and the chemokine receptor type 5 (CCR5), as well as obesity-specific genes known to activate pro-inflammatory mechanisms (56).

iNKT cells are reported to be highly expressed in AT of lean mice, and their abundance is reduced in the obese state (44). In human AT, iNKT cells are less abundant. AT-resident NKT cells have been demonstrated to modulate macrophage phenotype; IL-10-producing iNKT cells have been shown to trigger M2 polarization in steady-state AT. Further, iNKT-induced M2 polarization has been reported following acute or prolonged HFD challenges in an IL-4-dependent manner in mouse models and in human subjects with obesity (57, 58). In addition, AT-resident iNKT cells have been shown to trigger M2 macrophage polarization in the absence of HFD challenge in mice, via IL-10 (59). These results indicate that iNKT cells mitigate pro-inflammatory effects and induce metabolically favorable signaling in AT.

γδ T cells represent another subset of T cells that may act in AT (60). Usually, γδ T cells are prevalent in lean AT, but a further increase during obesity has been reported (61). Unlike the conventional CD4+ and CD8+ T cells, γδ T cells are negative for CD4 and CD8 markers, and express γδ T cell receptors (TCRs) (62). Further, the activation of these cells are mediated by TCR in a MHC-independent manner, and by receptors shared with NK cells (e.g., NKG2D and:DNAM-1) (63). The increase of infiltrating γδ T cells in AT in obesity has been shown to trigger the accumulation of ATMs, inflammation, and insulin resistance in mice, indicating that they are pro-inflammatory (61). In accordance with this finding, another study found reduced HFD-induced inflammation in AT of mice lacking γδ T cells (64). Nevertheless, further research is necessary to determine the exact role of γδ T cells is in obesity and obesity-related diseases.

NK cells and other innate lymphoid cells (ILCs) have been suggested as important mediators of the metabolic dysfunction of AT in obesity, in part through regulation of ATM activity. In AT of lean mice, NK cells were shown to exert homeostatic roles through killing M2-like ATMs, likely a mechanism to prevent them from turning pro-inflammatory (65). HFD promotes early accumulation of NK cells, especially in VAT, displaying reduced killing ability and increased IFNγ production that drive the polarization and maintenance of M1-like ATMs and development of insulin resistance (27, 66). Although less studied, AT NK cells in individuals with obesity were shown to display altered phenotype and function (67) (68), pointing to a similar role of these cells in human AT. However, the exact crosstalk between human NK cells and ATMs needs to be further elucidated.

Recently, DC has been found to play a crucial role in AT inflammation during obesity (69). DC expressing CD11c+ have been shown to be increased significantly in AT following HFD. Further, the DC increase in AT has been shown to be associated with CLS, thus confirming their association with ATMs. Importantly, a DC-null mouse model had a reduced number of AT macrophages, whereas DC replacement increased AT macrophage numbers in the DC-null mice. Finally, mice lacking DC did not gain weight or develop metabolic abnormalities when given an HFD (69). These data demonstrate that DC play an important role in systemic metabolic responses during obesity, and macrophage infiltration in AT.

Like other white blood cells, eosinophils are also involved in fighting disease and infections. In addition, eosinophils play a role in obesity that has become more evident in recent years. For example, the alternative activation of AT macrophages in obesity has been reported to be inextricably linked with eosinophils by an IL-4- or IL-13-dependent process (70). Briefly, IL-4 production by eosinophils was reported to promote thermogenesis in WAT, thereby increasing energy expenditure, limiting weight gain and improving glucose tolerance. In contrast, decreased eosinophil count was shown to be associated with increased weight gain and glucose intolerance in obese animals (70). Further, this study showed that M2-macrophages are greatly suppressed by the absence of eosinophils in WAT of obese mice. Similarly, eosinophils have been shown to control glucose homeostasis in people with obesity (71). These data suggest that eosinophils play a role in maintaining an anti-inflammatory ATM population to promote metabolic homeostasis.

Taken together, these studies confirm that in addition to ATMs, other immune cells are involved in metabolic inflammation and in the regulation of AT inflammation. These cells release proinflammatory cytokines which may directly affect metabolic pathways, but also modulate ATMs phenotypic characteristics to constitute a positive inflammatory feedback look. However, the role of macrophages and other immune cells during obesity is not limited to AT. We will now briefly discuss their role in other metabolic organs, such as the liver, pancreas, and gut.

## Obesity affects immune cells in non-adipose metabolic tissues

### Liver

The liver is important in a number of homeostatic functions, including detoxification, glucose metabolism, and synthesis of bile acids, proteins and lipids (72, 73). It is therefore noteworthy that obesity is a major cause of the most common chronic liver disease, non-alcoholic fatty liver disease (NAFLD), with recent data suggesting a world-wide prevalence of NAFLD of astonishing 40% (74). A sub-group of NAFLD patients develops chronic inflammation in the liver, which over time can lead to liver fibrosis, also known as non-alcoholic steatohepatitis (NASH) (1). The liver contains numerous immune cells, and accumulating evidence suggests that fatty acids and pro-inflammatory mediators released from AT may be important for liver immune cell activation and NASH progression, pointing towards the AT for a potential strategy to treat NASH (75). Several types of tissue resident immune cells (macrophages, neutrophils, B lymphocytes, T lymphocytes, and NK cells) have been reported to be associated with liver inflammation (76). The KC-liver macrophages constitute the largest population of liver immune cells and play an important role in NASH development (77, 78). They can be found within sinusoids, in contact with endothelial cells (79). Like ATMs, KCs can also be divided into pro- and anti-inflammatory M1/M2 subtypes (80), and the importance of KC polarization in the initiation and development of NAFLD and NASH is supported by several recent studies (81, 82). Additionally, KC subsets with distinct transcriptional profiles have been identified in NAFLD/NASH, reminiscent of heterogeneous ATMs in obesity. A recent study using scRNA-seq identified three distinct clusters of recruited macrophages (termed Mo-MFs) and a cluster of tissue resident KCs in HFD-fed mice (83). Another recent study, also using scRNA-seq, found distinct clusters of KCs in NASH livers. Two subsets of KC were identified by analyzing the levels of Cd5l expression and finally Trem2+ NASH-associated macrophages (NAMs) were identified by analyzing the levels of Trem2, Cd9, and Gpnmb expression in both mice and humans (84). These studies indicate that, like the AT, steatotic livers contain a heterogeneous pool of macrophages (84).

KC polarization may activate other immune cells in the liver that are relevant for obesity-related metabolic disease. One example is NK cells that normally participate in the defense against viral infections and tumor development, but that in the setting of obesity may promote fibrosis and NASH development (85). It has been shown that NK cell activity in the liver is stimulated by KCs (86). This is somewhat contrary to observations in AT, where inflammatory signals derived from NK-cells stimulate the pro-inflammatory polarization of ATMs (66). KCs have been shown to activate NK cells in the liver by generating IL-18 (87, 88), but also to suppress NK cell activity through the production of IL-10 that inhibits IFNγ expression and renders NK cells hyporesponsive in mice (86). Recent studies suggest that CD8+ T cells may also play important roles in liver immunopathology and NASH development (89, 90). However, there is still much to be discovered about the contribution of CD8+ T cells and their interaction with KCs under metabolic conditions leading to NASH. These findings indicate that KC polarization plays a major role in haptic inflammatory signaling during obesity and is thus involved in the development of NAFLD and NASH.

There has been little research on NK cells and inflammation in the liver during obesity. However, a recent study has reported that NK cells are responsible in inducing endoplasmic reticulum (ER) stress, and thus promote insulin resistance in obesity (91). In this study, HFD-fed mice display elevated production of proinflammatory cytokine osteopontin (OPN) in NK cells and this leads to ER stress and insulin resistance in the liver. At the AT level, OPN mediates macrophage infiltration, inflammation, and insulin resistance in mice during obesity. However, there is not yet a clear understanding of the role of liver NK cells derived OPN, and whether they are able to mediate inflammation in the liver by accumulating more macrophages.

Like in AT, the liver of obese (HFD) mice have been shown to be overpopulated with CD11c expressing DCs (69). This study also reported that increased liver DCs accumulated more macrophages, suggesting a macrophage-DC interaction and that DCs are involved in promoting liver inflammation during obesity (69). This is supported by human studies showing that DCs are critical in the development of liver fibrosis during obesity, thereby proving its involvement in liver inflammation (92, 93).

Overall, all these studies confirm that liver macrophages and their interaction with other liver immune cells are important mediators of metabolic dysfunction, and that targeting hepatic immune cells may represent a feasible strategy for the treatment of NASH and other metabolic disorders.

### Skeletal muscle

The skeletal muscle plays a significant role in metabolism. Normally, skeletal muscle is responsible for the majority of insulin-stimulated glucose disposal, suggesting that dysregulation of skeletal muscle metabolism can influence whole-body glucose homeostasis and insulin sensitivity (94). Indeed, obesity is associated with inflammation in myocytes/muscle cells, which may contribute to muscle inflammation. Nevertheless, changes in myocyte secretion of cytokines do not appear to constitute the major component of skeletal muscle inflammation in obesity. Instead, there is an increased accumulation of immune cells – in particular, macrophages and T cells - in the AT depots adjacent to the myocytes (95). This may explain why skeletal muscle immune cells in obesity resemble those identified in the AT, including the formation of CLS (96). AT resident immune cells may also help explain the fluctuations in the level of immune cell population and inflammation often observed in small-muscle biopsies, since this may be caused by variations in the ratio of muscle cells and AT (97). This may also explain why weight loss does not always seem to alter macrophage numbers in skeletal muscle (98).

Macrophages and T cells residing in skeletal muscles and their role in metabolic disorders have not been studied as extensively as in other tissues. One study showed that resveratrol alleviates obesity-induced skeletal muscle inflammation via decreasing M1 macrophage polarization and increasing the regulatory T cell population (99), suggesting that targeting these cell types may be of clinical importance. Studies exploring skeletal muscle macrophage activity are needed to explore the inflammatory pathway associated with obesity and comorbidities.

### Pancreas

The pancreas is essential for the regulation of macronutrient digestion. Maintaining metabolism/energy homeostasis via its exocrine and endocrine components, the pancreas controls blood sugar levels through insulin secretion and production in response to glucose intake (100).. The pancreatic immune system is composed mainly of macrophages and T cells (101, 102). Generally, pancreatic macrophages are present from embryonic development and shown to play a role in pancreatic islet morphogenesis and remodeling during the fetal and neonatal stages, but they may also be involved in adult pancreatic regeneration (103). However, the phenotypic characteristics of pancreatic macrophages remain enigmatic. Unlike ATMs and liver macrophage categorization, no M2 vs. M1 polarization paradigm has been suggested for metabolic regulation and dysfunction in islet macrophages. Healthy islets often have macrophages expressing M1 markers (CD11c, MHC-II), producing IL-1β, TNF-α, and expressing the pro-inflammatory transcription factor interferon regulatory factor (IRF)-5 (104–106). However, they do not express M2 markers (CD206), as compared to exocrine pancreatic stromal macrophages (105). It has been reported that a number of factors or stimuli in obesity can change the phenotype and function of islet macrophages, resulting in both acute and chronic pancreatitis (AP and CP). For example, like other tissue resident macrophages, islet macrophages have been shown to be activated by excess free fatty acids (FFA) (107). Further, infiltration of macrophages results in inflammation and tissue destruction in obesity, shown in islets from obese rodent models (88) and in T2D patients (89). Interestingly, caloric restriction and bariatric surgery have shown to diminish obesity-induced proinflammatory macrophage infiltration in the pancreas in experimental models (108), supporting the idea that pancreatic macrophages play a crucial role during obesity and obesity-induced pancreatic steatosis. Pancreatic macrophages have also been shown to be involved in autoimmune T1D. T1D occurs when the pancreatic beta cells lose their endocrine function, and genetically susceptible individuals develop a chronic lack of insulin in the system. Here, macrophages have been shown to accumulate autoreactive T cells, which leads to inflammation of the islets (“insulitis”) or immune-mediated degeneration of insulin-producing pancreatic beta cells. Overall, all this evidence suggests that, pancreatic macrophages, and T cells are the central mediators of low-grade chronic inflammation associated with obesity and T2D (109).

### Gut

Microbes that reside in the gut influence the host’s health directly through metabolic functions and immune system regulation. The innate immune system in the gut consists of a variety of cells, such as macrophages, ILC, DCs, and eosinophils, as well as epithelia. However, macrophages are the dominant innate immune cells in the gut, including a range of subtypes. At least five macrophage subpopulations were identified in mice and human intestine using flow cytometry (110). These subtypes have various functions, including maintenance of Tregs, crucial for responding to orally ingested antigens (111), and scavenging without causing inflammation. In obesity, it has been reported that there are significant changes in the gut-immune cell composition, leading to metabolic inflammation and gut dysbiosis via upregulation of inflammatory mediators like TLR4, TNF, and NFkB (112, 113). Gut dysbiosis enhances intestinal permeability by elevating the levels of gut bacterial lipopolysaccharides (LPS), which is then released into the systemic circulation to exacerbate low-grade inflammation and insulin resistance.

Among the gut-resident immune cells, increased levels of macrophages, a reduction of Tregs and increased Th1, CD8+, and γδ T cells have been reported in HFD-fed mice (114–116). In addition to these cells, ILC2s (produce proinflammatory IL-5 and IL-13) from the small intestine, have been also shown to be involved in the diet-induced obesity (117). A reduction in ILC3s (produce proinflammatory IL-17 and IL-22) levels in the lamina propria of HFD-fed mice has been shown to be correlated with reduced epithelial barrier integrity, increased serum LPS levels in gut, resulting in inflammation (115).

Overall, all these studies suggest that obesity-induced dysregulation of the gut metabolism may affect immune cell function, interaction, and composition, with some macrophage subtypes shown to play significant roles in obesity-induced metabolic diseases. These studies have revealed how microbiota affect systemic inflammation, suggesting that restoring gut microbiota could be a promising therapeutic target to treat obesity as an alternative to drugs and bariatric surgery.

## Small extracellular vesicles in cellular- and tissue crosstalk

Metabolic signaling between tissues occurs via neuronal connections and biochemical messengers in the circulation, such as adipokines, neuropeptides, chemokines, and cytokines. However, recent findings have highlighted that AT and other metabolic tissues also communicate via small extracellular vesicles (sEV) that are membrane particles derived from the cell (118). Because of their unique structure and physical characteristics, sEV have turned out to play a significant role in a variety of physiological processes, including immune responses, tissue repair and cell to cell communication (119). In addition, they are characterized by molecules biomarkers that are candidates for future diagnostic use in metabolic diseases like T2D (120).

sEV have a diameter of 30-200 nanometers and are constitutively produced from late endosomes and are secreted after fusion of multivesicular bodies (MVBs) with the plasma membrane. The cargo of sEV is composed of lipids, proteins, nucleic acids, organelles and other elements. There are several markers that confirm the exosome’s structural organization, including CD63, CD81, CD9, apoptosis-linked gene 2-interacting protein X (ALIX), heat-shock proteins (HSP60, HSP70, and HSP90) and tumor susceptibility gene 101 (TSG101). Commonly used non-specific sEV markers include glucose-regulated protein 94 (Grp94), calnexin (ER markers), GM130 (Golgi marker) and Cytochrome C (mitochondrial marker). These markers, which are not endogenously expressed on sEV, indicate that the sEV are released via an endocytic pathway and distinguish the sEV from necrotic bodies and other vesicles.

Recently, it has been reported that adipocyte-derived sEV exert a pivotal role in adipocyte-macrophage crosstalk during obesity (121). For example, adipocytes have been reported to release lipid-laden sEV (122) that express the lipid droplet-associated protein perilipin1 and carry phospholipids, neutral lipids, and free cholesterol that are taken up by ATMs to induce their differentiation. In another study, adipocytes from obese mice were found to release sEV with different mitochondrial composition than those derived from lean mice (123). This study also demonstrated that sEV cargo can control ATM activity and intra-organ transport of damaged mitochondria during metabolic stress.

It has been demonstrated that miRNA-containing sEV released by adipocytes influence ATMs phenotypes and function, as well as insulin sensitivity (124). For instance, it has been reported that rodents possess higher quantity of adipocyte-derived sEV-containing miR-34a during obesity (124, 125). This miRNA has been found to be interconnected with insulin resistance and metabolic inflammations by acting on ATMs. A study by Ying et al. (126) found that sEV enriched with miR-155 were mostly released from M1 macrophages isolated from obese AT. These miR-155 containing sEV have been shown to develop insulin resistance when injected into lean mice by directly targeting PPARg, leading to a reduced insulin expression and action. Besides acting as an inflammatory mediator, adipocyte-derived sEV may reduce inflammation, as demonstrated in adipocyte stem cells, and facilitate M2 macrophage polarization, thus offering a probable therapeutic intervention for obesity and metabolic disorders (127). Indeed, lean AT contained macrophages that emit sEV enriched in miR-690, were shown to improve systemic insulin sensitivity in obese mice (128).

**Figure 1:**
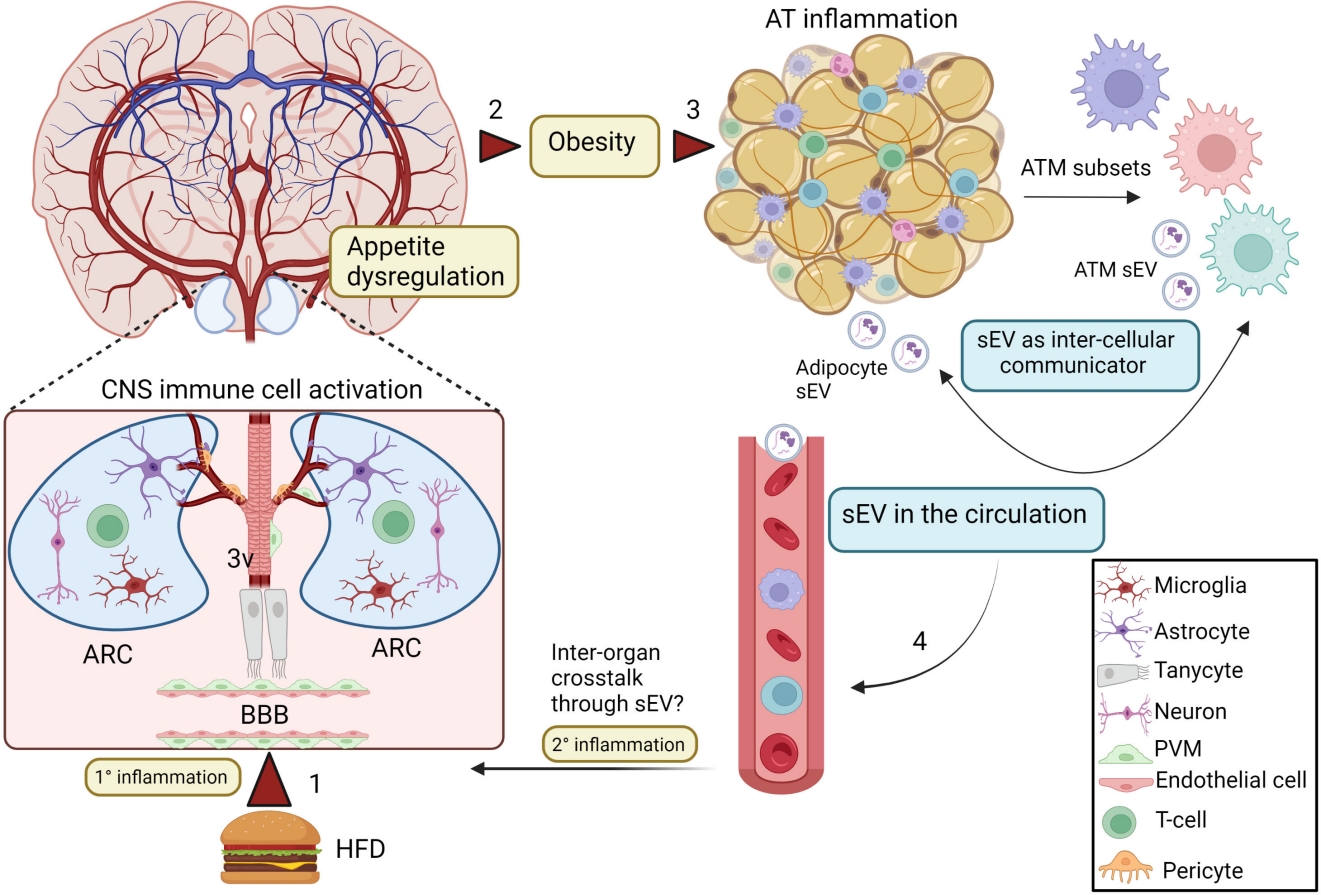
sEV as inflammatory mediators and cell to cell communicators in obesity. Excess nutrient intake may directly stimulate inflammation (1° inflammation) in the ARC (1), which leads to appetite dysregulation and obesity (2). This process involves activation of hypothalamic glial cells and neuronal interactions. In addition, tanycytes, that are special ependymal cells in the third ventricle of the brain (3V) are also involved in this inflammatory process in the CNS. Obesity also promotes inflammation and dysregulation of AT homeostasis (3), including increased macrophage infiltration and generation of various pro-inflammatory ATM subsets. During this altered microenvironment, both ATMs and adipocytes release sEV, which can mediate inter-cellular communication. AT-derived sEV can be released in the systemic circulation (4), but whether it can travel to the brain during obesity to participate in the secondary central inflammation from the circulation (2° inflammation) is still an open question. Created with BioRender.com. ARC, Arcuate nucleus; 3V, third ventricle; AT, adipose tissue; ATMs, adipose tissue macrophages; sEV, small extracellular vesicles; PVM, perivascular macrophage.

These reports confirm that sEV are important in cellular crosstalk within the adipose tissue. Further, these data support that obesity-induced inflammation is governed by the sEV/ATM axis, which not only provides mechanistic details of AT inflammation in metabolic disease, but also opens for a new approach to treating such diseases. Moreover, recent evidence suggests that AT derived sEV can be released into the circulation and target other major metabolic organs, such as the liver, the skeletal muscle, and the heart (121, 129). Still, there are relatively few studies that have investigated whether sEV mediate the connections between AT and the brain and influence central inflammation (130, 131) (**Figure 1**). Currently, researchers are also studying the role of native (132), stem cell-derived (127, 133), and bioengineered sEVs (134–136)in other metabolic tissues, which may lead to improved understanding of whole-body metabolic control and therapeutic approaches to obesity.

## Autonomic nervous system and macrophage crosstalk during obesity.

The autonomic nervous system (ANS) is a part of the peripheral nervous system that regulates involuntary physiological functions. ANS is further divided into the sympathetic, parasympathetic, and enteric nervous systems.

The innervation by sympathetic nervous system (SNS) of WAT was reported in the early 90s (137), and modulation mediated by SNS has been shown to influence weight changes in both rodents and humans through the release of norepinephrine (NE) (acting both as hormone and neurotransmitter) from sympathetic nerve endings (138–140). Macrophages have also been reported to release NE as well as expressing adrenergic NE receptors, and their activity can be affected by obesity-induced stress and the level of this neurotransmitter, suggesting a macrophage-SNS connection (141, 142). For instance, one study reported that anti-inflammatory ATMs release NE under cold stress (142), suggesting a role for these macrophages in the physiological response to reduced temperature. Other studies, however, reported that ATMs do not release NE (143, 144). A recent study shed new light on the connection between ATMs and NE: a cluster of sympathetic neuron–associated macrophages (SAMs) that reside in the AT was found to regulate obesity directly via the uptake and clearing of NE (145). This study demonstrated that SAMs act as a reservoir for NE and control NE uptake/elimination via the activation of the NE transporter solute carrier family 6 member 2 (SLC6A2) and the monoamine oxidase-A (MAO-A). Indeed, NE uptake by SAMs was blocked by genetic ablation of Slc6a2, which also led to increased browning of white fat, upregulation of thermogenesis, and weight loss in obese mice (145). Additionally, this study indicates that the molecular machinery encoding the NE clearance pathway is conserved across species, including humans, suggesting that obesity could be treated by targeting this pathway (145).

With regard to parasympathetic signaling, vagus nerve cholinergic signaling is an important major component. Vagus nerve activity regulates body weight and regulates inflammation in the GI tract and the liver. Ablation of vagus nerve signaling can promote hyperphagia and obesity, while vagus nerve stimulation promotes resolution of inflammation and obesity in mice through a mechanism that requires α7- acetylcholine (Ach) receptor (α7nAChR) subunit (146, 147). Intriguingly, it has been reported that macrophages, KCs, and DCs express 7nAChR, suggesting macrophage-vagus nerve crosstalk (148–151). For instance, peripheral activation of α7nAChR in mice has been shown to reduce NF-κB nuclear translocation and JAK2/STAT3 signaling, as well as lowering of proinflammatory cytokine production (152). This vagal nerve-macrophage signaling has also been reported to play a significant role in suppressing obesity-induced hepatic inflammation in mice models of NASH or NAFLD (153, 154). Similarly, in randomized clinical trials, modulation of this regulatory circuitry triggered by oral ingestion of acetylcholinesterase inhibitors has been shown to play a role in alleviating obesity-induced comorbidities in humans (155, 156), suggesting that modulating this circuitry may be an effective method of controlling obesity and obesity-related diseases. However, whether macrophages were involved in the manifestation of this effect remains unclear.

It is known that the enteric nervous system (ENS) controls intestinal motility, absorption of nutrients and whole body energy homeostasis (157). Interestingly, intestinal muscularis macrophages have been demonstrated to connect enteric neurons by releasing bone morphogenic protein 2 (BMP2, an osteogenic factor) which is required by enteric neurons for peristalsis to occur (158). As a result, the enteric neurons release the growth factor colony stimulating factor -1 (CSF-1) that supports the survival and function of enteric macrophages, confirming a symbiotic relationship. Also, it has been reported that the synapses between enteric neurons and muscularis macrophages express β2 adrenergic receptors (159), further underlining a relationship between macrophages and the peripheral nervous system. Thus, any changes in metabolism during obesity could negatively affect intestinal motility, nutrient absorption, and energy metabolism. For example, gastrointestinal alterations, mainly dysmotility, have been reported at the onset of obesity or in HFD animal models (160, 161). Further, in response to bacterial stimulation, NE was shown to be released by sympathetic neurons and to stimulate adrenergic signaling, which in turn activates a macrophage response in the intestine (159). This indicates that gut microbiota changes caused by obesity or diet changes may affect the relationship between enteric neurons and macrophages.

In view of the observations made above, macrophages can actively interact with peripheral nervous system in obesity, suggesting that modulating peripheral nervous system-macrophage axis may be useful in treating obesity in the future.

## Central nervous system inflammation during obesity: role of macrophages and other immune cells

So far, we have focused on how activation of peripheral tissue immune cells, in particular macrophages, may impact organismal metabolic control. However, the obesity-induced central inflammation is more complex. Although it is becoming increasingly clear that microglia, which are specialized macrophages in the CNS, may contribute to obesity-associated metabolic changes (162), recent evidence also indicate that this central inflammation is influenced by the activity of other CNS cells, such as astrocytes and tanycytes, and their interactions with neurons (163, 164).

In particular, the microglial cell population located in the mediobasal hypothalamus (MBH), an anatomically distinct brain region that includes the arcuate nucleus (ARC) and median eminence, can be activated to a pro-inflammatory phenotype in response to nutrient excess (165, 166). For example, palmitic acid (PA)-induced neuro-inflammation has been shown to involve microglial activation (167, 168). Interestingly, the deactivation of FABP4 in microglia has been shown to decrease the PA induced inflammation by expressing more microglial UCP2 (169), indicating that overnutrition can trigger microglial activation, which may exacerbate hypothalamic inflammation.

It is well known that the hypothalamic neuronal population in the arcuate nucleus (ARC) controls metabolic feedback and regulates energy homeostasis in the body (170–172). Two functionally antagonistic neuronal populations contribute to this regulatory function; one cluster of neurons that expresses the orexigenic neuropeptides agouti-related peptide (AgRP) and neuropeptide Y (NPY), and a second cluster that expresses the anorexigenic peptides proopiomelanocortin (POMC) and cocaine and amphetamine regulated transcript (CART). Interestingly, the activity of these neurons has been reported to be altered by microglial activation during obesity (173). For instance, microglial activation increased TNFα secretion, leading to mitochondrial stress and altered firing rates in adjacent POMC neurons, thus contributing to the development of obesity in mice (174). On the contrary, increased exercise levels in mice led to reduced hypothalamic microglial activation and improvement of glucose tolerance, suggesting that microglial inflammation may be a potential therapeutic target to ameliorate metabolic dysfunction (175).

Microglia are derived from yolk sac and usually do not get replaced by circulating progenitors derived from the bone marrow (176). Nevertheless, during obesity, microglia-like myeloid cells from the circulation may infiltrate the MBH to be engaged in inflammatory crosstalk with the resident microglia (177). It may be difficult to separate the different myeloid cell populations in the brain, but cell surface markers to define distinct microglial phenotypes have been reported (178). Interestingly, it was shown that a positive energy balance in the form of high glucose treatment triggered the expression of many of the pro-inflammatory markers on microglial cells recognized from peripheral macrophages, including ionized calcium-binding adapter molecule 1 (Iba1), CD68, high mobility group box-1 protein (HMGB1), and CD11b (179), whereas the expression of anti-inflammatory microglial markers, such as CD206 and arginase-1, were decreased. Further, mimicking the environment of diabetes (characterized by hyperglycemia and hyperlipidemia) in cultured microglial cells, supplement with high glucose and free fatty acids (mixture of palmitic and oleic acids) was shown to promote Iba1 and CD11b expression, induce microglial morphological changes, raise oxidative stress levels, and stimulate IL-1 production and TNF production. This indicates that elevated levels of circulating nutrients may change microglial activity and phenotype to cause central inflammation, which may lead to dysregulation of feeding behavior in the *in vivo* setting.

Microglial activity can also be modulated by circulating nutrients, neuropeptides and hormones that are altered in obesity (180). The POMC-derived peptide alpha melanocyte stimulating hormone (α-MSH) and hormones like ghrelin and leptin have gained attention due to their ability to reduce or induce inflammation in the brain (181–184). Fatty acids and leptin are both elevated in the obese state and can promote cytokine secretion from microglia through activation of the TLR4/IKK/NF-κB pathway. Leptin has also been shown to induce NF-κB through the LepR/IRS1/AKT pathway (185, 186). In addition to hypothalamic inflammation, altered microglial homeostasis in the hippocampus and amygdala has been reported during HFD feeding in mice, although the activation of microglia in these areas mainly affects cognitive performance (187).

Other than parenchymal macrophages or microglia, non-parenchymal macrophages, including perivascular macrophages (PVMs), choroid plexus macrophages, and meningeal macrophages, inhabit the interface between the brain and the periphery where they function as “guardians”/”guards” against invading microorganisms and suppress harmful inflammation (150). PVMs are considered important defense immune cells, but with dual physiological functions (188). On the one hand, PVMs has been shown to mediate activation in the HPA axis by prostanoids (lipid mediators that stimulate the inflammatory response) (188, 189), whereas on the other hand PVMs have been shown to limit endothelial involvement in inflammatory processes, thereby inhibiting CNS responses to inflammatory insults. Nevertheless, during obesity PVMs seem to mostly be involved in pro-inflammatory activation. Elevated saturated fatty acid (SFA) levels and inflammatory molecules in the circulation have been shown to activate PVM signaling molecules, such as nitric oxide (NO), which may mediate increased vascular permeability and accumulation of lipids in the hypothalamus (3, 151). Vascular endothelial growth factor (VEGF) is another signaling molecule expressed by PVM. Elevated serum VEGF level is associated with obesity, poor glycemic control, and T2D (190, 191). Interestingly, the expression of VEGF in PVMs has been reported to be increased during the first four weeks of HFD feeding in mice, a period during which no significant increase in the expression of VEGF is evident in the spleen, liver, or AT (192). This suggests PVMs as a primary source of this pro-inflammatory growth factor. The PVMs may also have an effect on microglial cell function during obesity, and it was reported that PVMs, together with meningeal macrophages, may induce microgliosis in the hypothalamus (177). Indeed, the number of meningeal macrophages is elevated during HFD, indicating that these cells can trigger diet-induced microgliosis (177). The choroid plexus macrophages have also been shown to play critical roles in brain inflammation, but this seems to occur during neurodegenerative diseases such as Alzheimer’s disease, neurodevelopmental and psychiatric disorders (193–195). The role of these cells in inflammation induced by HFD and obesity is still elusive.

The astrocytes are another type of glial cell found in the CNS, and normally they are responsible for supporting differentiation and homeostasis in neurons, as well as influencing synaptic activity. However, during HFD induced obesity, astrocytes have been reported to interact with microglia and augment hypothalamic inflammations (196, 197) probably due to an increase in astrocytic NF-κB signaling (198), or due to increased chemokine signaling in microglia. For instance, conditioned medium of palmitate-treated reactive astrocytes has been shown to increase the expression of microglial chemokine CCL2, which promotes migration of microglia (196), supporting that interactions between astrocytes and microglia may contribute to obesity-induced inflammation in the CNS.

Taken together, these studies confirm that central immune cells, especially microglia, play a major role in obesity-induced central inflammation. Although the brain contains other immune cells such as T cells, B cells, and NK cells, their role and interaction with microglia and astroglia remain unexplored. Central inflammation affects appetite regulation, and thus peripheral metabolism through increased food intake and obesity **Box 1**. Further research on the interactions between CNS- and peripheral immune cells and between different types of brain-resident immune cells in a context of obesity is needed to better understand the how obesity-related central inflammation affect whole-body metabolism.

## Concluding remarks

Tissue-resident, pro-inflammatory immune cells give rise to systemic inflammation and play a role in the development of metabolic dysregulation associated with obesity. The purpose of this review has been to shed light on peripheral and central immune cells in obesity and obesity-related disorders, with particular focus on macrophages. Macrophages are characterized by significant diversity and plasticity both within and between tissues. Their phenotypes are modulated by intricate communication with other immune cells as well as with parenchymal cells, such as adipocytes and neurons. During obesity, inflammation in peripheral tissues leads to release of pro-inflammatory cytokines into the circulation, from where they may cross the BBB to further activate brain resident macrophages and stimulate inflammation in the CNS. However, hormones, neuropeptides, and dietary fatty acids have also been shown to change macrophage function in the brain directly, independent of peripheral inflammation. Adding another layer of regulation are the sEVs, which are particularly interesting for their involvement in inter-organ communications. The adipocyte/ATM axis is important for obesity-induced inflammation and the involvement of sEV during this process seems to be of great importance. The contribution of sEVs to the CNS inflammation is also an interesting area of research that needs to be further pursued.

## Future perspectives

The development of anti-obesity therapies has proved to be extremely challenging from a methodological, technical, and societal perspective. Therefore, in-depth research into neglected areas can lead to new anti-obesity therapies that can benefit medical fields and society. For example, when it comes to the role of central macrophages in obesity and CNS inflammation, most studies have focused on microglia. However, other macrophage subsets may also be of relevance, such as those in the perivascular space, meninges, and choroid plexus that have so far been mostly neglected. Some studies have shown their connection to hypothalamic inflammation during diet-induced obesity, but their exact role in hypothalamic inflammation remains unclear, and more research is needed to understand how these non-parenchymal macrophages contribute to obesity-induced inflammation and if they affect regulation of food intake or energy expenditure.

Exploiting the properties of sEV is a promising strategy for targeted delivery of molecules to specific CNS cell types, potentially leading to more specific clinical effects and reduced side-effects. Efforts in this field should aim to understand both basic-mechanistic concepts as well as translate them into clinical practice. Due to the extremely complex nature of obesity-related inflammatory activation, involving numerous organs, cell types, signaling pathways and feedback mechanisms, the distance from “bench to bedside” appears long. However, in the face of the escalating global obesity epidemic, the pursuit of novel therapeutic agents, resting on sound fundamental research, should be continued.

**BOX 1: Can peripheral and central inflammation be affected by obesity in a bidirectional manner?**

**Figure 2:**
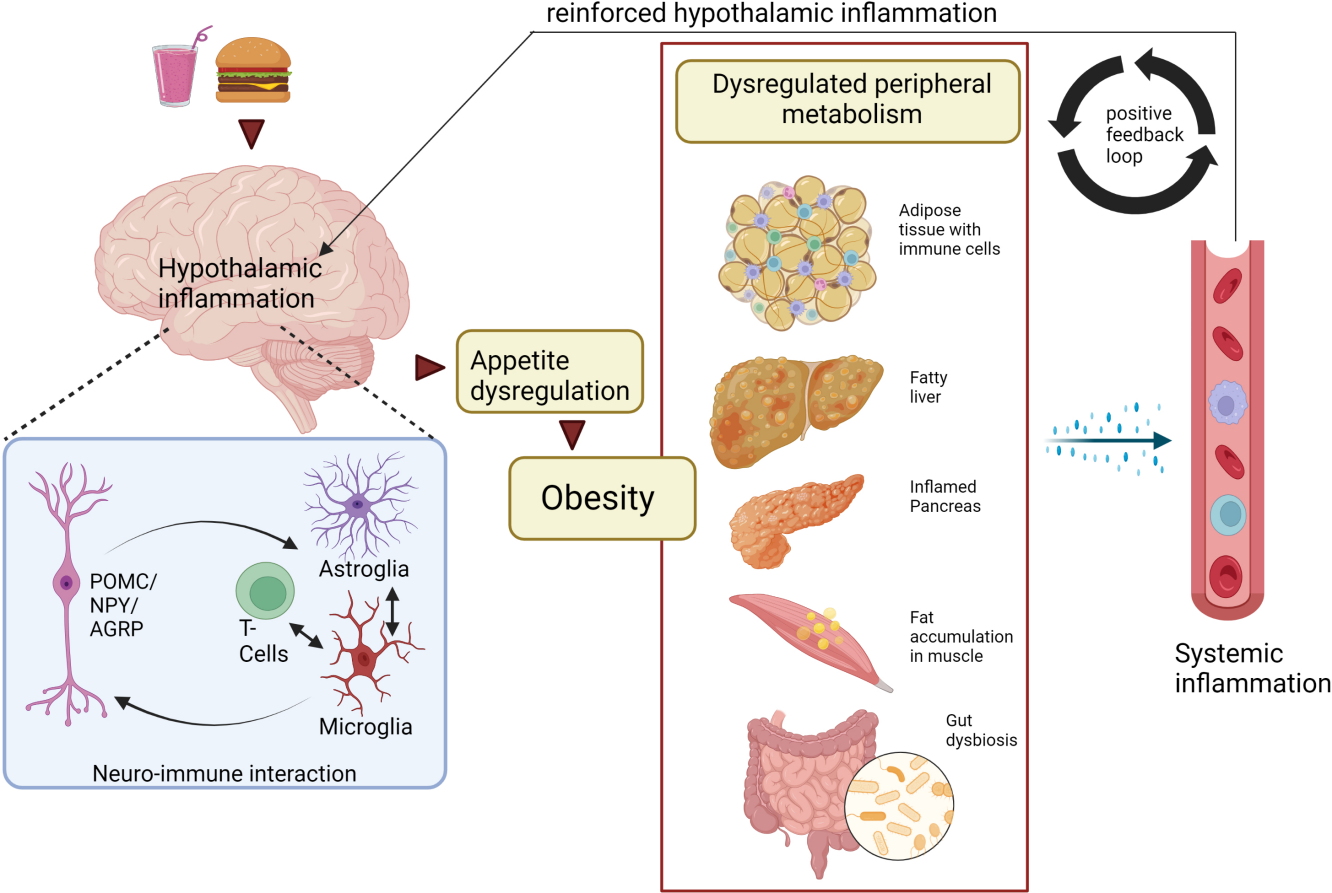
Peripheral and central macrophage interaction during obesity: a bidirectional mechanism? Neuro-immune interactions trigger hypothalamic inflammation and obesity. As a result, tissue-resident macrophages and other immune cells in AT, liver, pancreas, muscle, gut are activated, thereby dysregulating metabolism within these peripheral tissues. Furthermore, the tissue-resident immune cells release proinflammatory cytokines and chemokines into the circulation, some of which can enter the brain and exacerbate hypothalamic inflammation though the activation of glial cells (especially microglia) and tissue resident T-cells. Ultimately, this will result in sustained CNS inflammation and obesity, suggesting a bidirectional interaction between CNS and peripheral inflammation. Created with BioRender.com. CNS, Central Nervous system; AT, adipose tissue; POMC, Pro-opiomelanocortin neurons; NPY, Neuropeptide Y neurons; AgRP, Agouti-related protein.

Several studies have indicated that during obesity, peripheral tissues and the CNS are in constant communication with each other, and that this signaling includes inflammatory mediators (199–201). However, it has proven difficult to identify the origin of the inflammatory signal. On the one hand, inflamed AT has been shown to send pro-inflammatory, adipokine-mediated signals to the hypothalamus, which then controls energy homeostasis, feeding behavior, and metabolic rate. Also, circulating myeloid cells can be recruited to the CNS and subsequently be involved in hypothalamic inflammation that contribute to further food intake dysregulation (177). On the other hand, it was shown that inflammatory signaling in the hypothalamus occurred in response to nutrient overload before weight gain occurred and peripheral inflammation had taken place, suggesting that peripheral inflammation is rather a consequence of obesity than the source of an initial stimulating signal to the CNS (177, 202). Thus, the inflammation in the peripheral tissues that is relevant for systemic insulin sensitivity and metabolic dysfunction seems to be secondary to obesity, and that hypothalamic inflammation and dysregulation of food intake is the primary signal. Nevertheless, a positive feedback loop from the periphery to the CNS seems to be important in maintaining obesogenic signaling (**Figure 2**) (177, 203).

The ability of the peripheral tissues to communicate metabolic status to the CNS is of great importance. One example is the gut hormone glucagon-like peptide-1 (GLP-1) that is released into the bloodstream after feeding (204, 205). In general, it stimulates insulin secretion by potentiating the insulinotropic effects of glucose, and it also reduces appetite by acting in the hypothalamus (206). The beneficial effects of GLP-1 have been exploited pharmacologically, and there is currently a surge in GLP1R analogues and other gut hormone-based therapies that is becoming available for clinical use. Interestingly, gut hormones may also affect CNS inflammation; dysbiosis in the gut during obesity has been shown to cause the activation of hypothalamic glial cells (especially microglia) through abnormal GLP-1 receptor (GLP-1R) signaling (207). The GLP-1R signaling has also been established in Tanycytes (a special type of ependymoglial cell located near the third ventricle), and they have been reported to be involved in obesity-related CNS inflammation. Blocking GLP1R in tanycytes has been shown to prevent the transport of the anti-diabetic drug and GLP1R agonist liraglutide into the brain and its activation in the hypothalamic neurons, as well as its anti-obesity effects on food intake and body weight (208). This shows the potential of glial-like cell types in the hypothalamus to modulate GLP-1R signaling from the gut and targeting this signaling could develop new treatments for obesity. Weight loss surgery further emphasizes gut-macrophage-neuron crosstalk and the importance of bidirectional communication between the CNS and the periphery. For example, Roux-en-Y gastric bypass (RGYB) has been shown to alter the interaction between microglia and POMC neurons due to alteration in circulating humoral factors and leads to improvement in hypothalamic inflammation (irrespective of weight loss) and leptin sensitivity, suggesting a CNS-periphery crosstalk (209). In respect to humoral factors, the sEV could serve as promising candidates for a CNS-targeted strategy in the treatment of obesity. sEV can be loaded with specific cargos (e.g. lipids, proteins, DNA, and RNA) that can be transported in the circulation and targeted to specific cell types in the hypothalamus, which in turn can modulate CNS inflammation and ameliorate whole body metabolism. These possibilities emphasize the importance to understand more about how inflammation and immune cell are involved in the cross talk between the periphery and the brain, insight that will be of uttermost important in the quest for future therapies for obesity and metabolic disease.

## Author contributions

SM drafted the article which was critically reviewed by all authors during the development of the article. All authors listed have made a substantial, direct and intellectual contribution to the work, and have approved it for publication.
